# Baseline molecular surveillance of monkeypox virus in wildlife and exotic animals during the pre- and early-2022 outbreak in Thailand

**DOI:** 10.14202/vetworld.2026.1504-1520

**Published:** 2026-04-24

**Authors:** Tatiyanuch Chamsai, Natthaphat Ketchim, Metawee Thongdee, Somjit Chaiwattanarungruengpaisan, Ladawan Sariya, Supakarn Kaewchot, Sarin Suwanpakdee, Weena Paungpin, Nattarun Chaisilp

**Affiliations:** 1 The Monitoring and Surveillance Center for Zoonotic Diseases in Wildlife and Exotic Animals, Faculty of Veterinary Science, Mahidol University, Nakhon Pathom 73170, Thailand; 2 Department of National Parks, Wildlife and Plant Conservation, Chatuchak, Bangkok 10900, Thailand; 3 Department of Clinical Sciences and Public Health, Faculty of Veterinary Science, Mahidol University, Nakhon Pathom 73170, Thailand

**Keywords:** animal surveillance, exotic pets, molecular detection, monkeypox virus, One Health, real-time PCR, wildlife reservoirs, zoonotic spillback

## Abstract

**Background and Aim:**

The global outbreak of monkeypox (mpox) in 2022 raised major concerns regarding the potential establishment of new animal reservoirs outside endemic regions. Although human-to-human transmission was the primary driver of the outbreak, reverse zoonotic transmission (spillback) from infected humans to animals could lead to long-term viral persistence in wildlife populations. Thailand, a non-endemic country affected during the 2022 outbreak, has extensive human–animal interfaces involving free-ranging wildlife, exotic pets, and urban scavenging mammals, which may facilitate cross-species transmission. The present study aimed to perform molecular surveillance for mpox virus (MPXV) DNA in selected high-risk animal populations in Thailand using archival samples collected during the pre-outbreak (2019) and early-outbreak (2022) periods to establish baseline evidence of viral presence or absence.

**Materials and Methods:**

A total of 1,248 animals, including 593 free-ranging cynomolgus macaques (*Macaca fascicularis*), 436 exotic pets, and 219 garbage-scavenging small mammals, were included in this cross-sectional surveillance study. Swab samples (oral, nasal, buccal, or rectal) collected during 2019 and 2022 were retrieved from archival storage. Viral DNA was extracted using a commercial genomic DNA kit, and detection of MPXV was performed by real-time polymerase chain reaction targeting the *B7R* gene, a conserved region specific to MPXV. Samples were pooled in groups of 4–5 to increase screening efficiency. Descriptive statistics were used to determine prevalence, and the rule-of-three method and binomial probability model were applied to estimate confidence limits and surveillance power in the absence of positive results.

**Results:**

All 1,248 samples tested negative for MPXV DNA. The studied population represented 29 animal species across multiple ecological settings, including urban, peri-urban, and rural environments in several provinces of Thailand. Based on the sample size, the upper 95% confidence limit for prevalence was estimated to be <0.24%. Power analysis indicated a 99.81% probability of detecting at least one positive case if the true prevalence had been ≥0.5%, confirming strong surveillance sensitivity.

**Conclusion:**

This study provides the first ecological baseline evidence of the absence of MPXV in wildlife, exotic pets, and garbage-scavenging mammals in Thailand before and during the early phase of the 2022 global outbreak. The findings support the importance of One Health-based surveillance integrating wildlife, domestic animals, and environmental interfaces to prevent spillback events and the establishment of new reservoirs in non-endemic regions. Continued longitudinal monitoring using both molecular and serological approaches is essential for early detection of emerging zoonotic threats.

## INTRODUCTION

Monkeypox (mpox) is an infectious viral disease caused by mpox virus (MPXV). MPXV is a double-stranded DNA virus of ~200 kb genome size that belongs to the genus *Orthopoxvirus* (OPXV) of the family *Poxviridae* [1, 2]. In the OPXV genus, four species are significant human pathogens: variola virus (VARV), which causes smallpox; MPXV; cowpox virus (CPXV); and vaccinia virus (VACV) [3, 4]. MPXV and VARV are the most virulent pathogens within the genus [5, 6]. MPXV has been categorized into two clades based on genomic studies: clade I (formerly the Central African, Congo Basin clade) and clade II (formerly the West African clade). These two clades differ geographically in epidemiological and clinical characteristics. The Central African clade tends to be more pathogenic than the West African strains [7–9]. MPXV is transmitted to humans through direct contact with lesions, body fluids, or respiratory droplets from either an infected animal or an infected person [10, 11].

MPXV was first isolated in 1958 in Denmark from laboratory cynomolgus macaque (*Macaca fascicularis*) with a pox-like disease [12]. Human mpox was first identified in 1970 in the Democratic Republic of Congo (DRC) [12, 13]. Sporadic human infections with MPXV have been reported in Central Africa with a mortality rate of up to 10%, whereas West Africa has a case fatality rate of approximately 1%, with a higher mortality rate in individuals co-infected with human immunodeficiency virus (HIV) [14, 15]. In 2022, the largest mpox outbreak occurred, affecting more than 140 countries globally, of which at least 115 countries had never previously reported mpox [16]. The total number of confirmed cases reached 163,355, including 426 deaths, from 1 January 2022 to 30 September 2025, according to the World Health Organization (WHO) [16]. The Region of the Americas reported the highest cumulative number of cases (70,451 cases with 154 deaths), followed by the African region (53,647 cases with 227 deaths) and the European region (30,765 cases with 10 deaths), as of September 2025 [16].

Although mpox is a viral zoonosis, the exact natural reservoir host remains unidentified despite the ability of MPXV to infect a wide range of mammalian species. Evidence of MPXV in wild animals under natural conditions is limited. The virus has previously been isolated or detected from wildlife hosts mainly in endemic areas. MPXV has been isolated twice in history, first in 1985 from a Thomas’s rope squirrel *Funisciurus anerythrus* in DRC and later in 2012 from a sooty mangabey Cercocebus atys in Ivory Coast [17, 18]. In addition, MPXV DNA has been detected in museum specimens of African rope squirrels (*Funisciurus anerythrus*, *Funisciurus carruthersi*, *Funisciurus congicus*, *Funisciurus lemiscatus*, and *Funisciurus pyrropus*) collected across Central Africa [19], and viral DNA has also been observed in feces of chimpanzees *Pan troglodytes* verus in Ivory Coast [20]. Nevertheless, rodents are believed to be the most likely reservoirs of MPXV [21, 22]. Antibodies against MPXV have been detected in 2 out of 18 squirrels tested in DRC [17]. Moreover, squirrels of the genera *Funisciurus* and *Heliosciurus*, rodents of the genera *Cricetomys* and *Graphiurus*, and elephant shrews of the genus *Petrodromus* have been linked to the natural cycle of MPXV in DRC [23–26].

Previously, outbreaks of mpox occurred only in endemic regions of Africa, where disease incidence increased, possibly due to human encroachment into animal habitats [25–27]. However, the first detection of mpox in humans outside Africa occurred in the western hemisphere during an outbreak in the Midwest of the United States between April and June 2003 [28]. This outbreak was linked to the importation of African rodents, including giant pouched rats *Cricetomys* spp., rope squirrels *Funisciurus* sp., and African dormice *Graphiurus* sp., from Ghana to the United States for the pet trade [29]. Investigations revealed that several native animal species sharing bedding and cages with the imported rodents were infected with MPXV, including black-tailed prairie dogs *Cynomys ludovicianus*, hedgehogs *Atelerix* sp., southern opossums *Didelphis marsupialis*, gray short-tailed opossums *Monodelphis domestica*, jerboas *Jaculus* sp., and woodchucks *Marmota monax* [29]. Among these, infected prairie dogs sold as pets were identified as the source of human infection during the outbreak [30, 31].

Thailand is one of the non-endemic countries affected by the largest global MPXV outbreak in 2022. As of September 2025, Thailand has reported more than 930 confirmed human mpox cases, including 13 deaths [16, 32]. The emergence of mpox in several non-endemic countries, including Thailand, occurred through exportation of MPXV from endemic regions or travel-associated cases. Undetected transmission within surveillance systems for an unknown period, followed by recent amplification events, may have contributed to the expansion of the outbreak in affected regions [33]. Although the spread of the virus during the current outbreak has been mainly driven by human-to-human transmission through intimate or sexual contact, the risk of viral spillback from humans to animals remains a major concern. Animal populations at high-risk of infection, particularly those living in close contact or proximity to infected humans, must be monitored to prevent the establishment of new animal reservoirs and their maintenance between outbreaks [34]. Additional potential animal populations also require investigation.

Mitigating the spillback risk of MPXV in non-endemic regions is a critical public health priority to prevent the establishment of permanent enzootic cycles in susceptible local host species [35]. The formation of such reservoirs could lead to recurrent human outbreaks. In novel animal hosts, MPXV may undergo genetic adaptation, potentially giving rise to variants with increased transmissibility or altered pathogenicity [36]. Furthermore, frequent asymptomatic infections in animal populations may allow cryptic viral circulation, complicating surveillance and undermining global strategies for early detection and containment [37].

Despite the global spread of mpox during the 2022 outbreak, information regarding the presence of MPXV in animal populations outside endemic regions remains extremely limited. Most previous investigations have focused primarily on human epidemiology, whereas data on potential wildlife, exotic pet, and urban scavenging animal reservoirs in non-endemic countries are scarce. The possibility of reverse zoonotic transmission (spillback) from infected humans to animals has raised concern that MPXV could establish new enzootic cycles in susceptible host species, particularly in countries with extensive human–animal interaction. Thailand represents a high-risk interface because of the large population of free-ranging macaques, widespread ownership and trade of exotic pets, and the presence of synanthropic small mammals living in close proximity to human communities. However, no nationwide molecular surveillance has been conducted to determine whether MPXV was present in animal populations before or during the early phase of the global outbreak. In addition, the lack of baseline data collected prior to the 2022 outbreak limits the ability to distinguish newly introduced infections from pre-existing silent circulation of MPXV. Therefore, systematic surveillance using archival and outbreak-period samples from high-risk animal populations is essential to provide an ecological baseline and to evaluate the potential risk of reservoir establishment in non-endemic regions.

Therefore, the present study aimed to investigate the presence of MPXV in selected high-risk animal populations in Thailand using a cross-sectional molecular surveillance approach. Archival and outbreak-period swab samples collected from free-ranging cynomolgus macaques, exotic pets, and garbage-scavenging small mammals were examined for MPXV DNA using real-time PCR targeting the *B7R* gene. Samples collected during the pre-outbreak period (2019) and early-outbreak period (2022) were included to establish baseline evidence of viral presence or absence and to assess the potential for cryptic circulation prior to widespread human transmission. By integrating wildlife, exotic animal, and environmental interface populations, this study was designed within a One Health framework to evaluate the risk of spillback transmission and to provide reference data for future surveillance programs aimed at preventing the establishment of permanent MPXV reservoirs in non-endemic regions.

## MATERIALS AND METHODS

### Ethical approval

The use of archival animal samples in this study was reviewed and approved by the Institutional Animal Care and Use Committee of the Faculty of Veterinary Science, Mahidol University (Protocol Number: MUVS-2022-07-44).

The study protocol involving MPXV handling and molecular testing was reviewed and approved by the Institutional Biosafety Committee of Mahidol University (Protocol Number: MU 2022-022).

All laboratory procedures involving primary specimens were conducted within a certified Class II biological safety cabinet in a biosafety level 3 facility. Staff adhered to stringent safety protocols, including the use of double gloves and N95 respirators, and initial sample lysis for DNA extraction was performed to ensure viral inactivation before downstream PCR analysis.

### Study period and location

Swab samples from free-ranging *M. fascicularis* were collected between March and November 2019 (pre-outbreak) across 15 provinces in Thailand, covering the Central (Bangkok, Lopburi, Saraburi, Nakhon Sawan, and Phetchabun), Northeastern (Amnat Charoen, Maha Sarakham, and Mukdahan), Eastern (Chon Buri), Western (Phetchaburi and Ratchaburi), and Southern (Krabi, Narathiwat, Phatthalung, and Satun) regions. Samples from exotic pets and small mammals were collected between May and September 2022 (early-outbreak) in Bangkok, Nakhon Pathom, and Nonthaburi.

For site selection and sampling criteria, provinces were selected for wildlife and exotic animal surveillance based on their status during the 2022–2024 global outbreak to capture a range of exposure risks [16]. Mpox-affected areas were defined as provinces with high human case numbers or significant international travel hubs, including Bangkok (representing approximately 90% of national cases), Nonthaburi, Chon Buri, and a major southern tourist destination such as Krabi [32]. In contrast, non-affected or low-incidence areas were selected across the Northeastern (Amnat Charoen, Maha Sarakham, and Mukdahan), Western (Phetchaburi and Ratchaburi), and inland Central regions (Nakhon Pathom, Lopburi, Saraburi, Nakhon Sawan, and Phetchabun) to provide a comparative baseline of viral absence in animals away from primary transmission hotspots. This dual selection strategy ensured that the ecological baseline covered both high-risk zones for potential human-to-animal spillback and lower-risk rural environments.

In terms of urban and climatic context, the study provinces represented a diverse range of urbanized and natural landscapes within Thailand’s tropical monsoon climate. Urban and peri-urban hubs, including Bangkok, Nonthaburi, Nakhon Pathom, and Chon Buri, are characterized by high human density and fragmented green spaces where wildlife, such as urban rodents and macaques, frequently interact with human populations [38, 39]. In addition, central provinces, including Lopburi and Saraburi, are characterized by significant human–wildlife overlap, particularly with non-human primate populations in urban centers [40]. In contrast, provinces in the Northeastern (Amnat Charoen, Maha Sarakham, and Mukdahan), Western (Phetchaburi and Ratchaburi), Southern (Krabi, Narathiwat, Phatthalung, and Satun), and Central (Nakhon Sawan and Phetchabun) regions consist primarily of rural, agricultural, and mountainous landscapes with higher forest cover, providing distinct ecological settings for potential wildlife reservoirs [41]. While the Central and Eastern regions experience significant temperature fluctuations between the hot and cool seasons, the Southern coastal provinces maintain a more stable, humid tropical climate year-round. Unlike the humid coastal South, the Northeastern provinces experience lower relative humidity and higher peak temperatures during the dry months [42]. This selection accounts for varying ecological factors that influence both the persistence of MPXV in the environment and the risk of spillback into local wildlife reservoirs.

### Animal selection criteria

The selection of target animal species was based on specific criteria derived from current scientific literature and ecological risk assessment.

Inclusion criteria were based on two primary ecological and biological factors:

1. Potential reservoir susceptibility: Species were selected based on evidence from previous mpox outbreaks or experimental studies that identified them as known or suspected reservoirs or susceptible hosts, such as various rodent species and non-human primates.
2. Proximity to human settlements: Targeted animals included free-ranging populations living in close proximity to or sharing habitats with human communities, such as urban macaques and peri-urban rodents.

Based on these criteria, the study focused specifically on free-ranging macaques, small mammals, and exotic pets.

Exclusion criteria included livestock and common domestic animals, such as dogs and cats. These species were excluded from this surveillance effort because they are less likely to serve as primary maintenance reservoirs for MPXV than the targeted wildlife and exotic species during the study period.

### Species level identification methods for small mammals

Small mammals were identified to the species level primarily through morphological assessment. Field identification followed standard taxonomic keys for Southeast Asian rodents, utilizing external morphometric parameters including body weight, head-and-body length, tail length, condylobasal length, ear length, and hindfoot length [43, 44]. To maintain taxonomic accuracy, immature specimens with ambiguous external features, due to the unreliability of morphometrics in juvenile rodents, were conservatively recorded as unidentified rat species.

### Sample types and collection procedures

Archival swab samples used in this study were categorized into three primary animal groups based on their ecological role and the timing of collection:

1. **Free-ranging macaques (pre-outbreak baseline):** A total of 593 *M. fascicularis* were sampled from 24 free-ranging colonies across 15 provinces between March and November 2019. These pre-outbreak samples were obtained through archival buccal, oral, oropharyngeal, or rectal swabs. Sampling was conducted in collaboration with the Department of National Parks, Wildlife and Plant Conservation (DNP) (Table 1; Supplementary Table S1).
2. **Exotic pets (early-outbreak surveillance):** To assess potential accidental reservoirs during the early phase of the 2022 global outbreak, 436 exotic pets were sampled between May and September 2022. Nasal, oral, or rectal swabs were collected from various species presented to animal hospitals in Bangkok and in the adjacent province (Nakhon Pathom). Sampling was conducted under the SARS-CoV-2 research project supported by Health Security Partners, the U. S. CDC, and the Thailand MoPH-U. S. CDC Collaboration (Table 2; Supplementary Table S2).
3. **Garbage-scavenging small mammals (early-outbreak surveillance):** A total of 219 small mammals, including species known for garbage-scavenging behavior, were sampled between May and September 2022 at garbage dumping sites in Bangkok and two adjacent provinces, Nakhon Pathom and Nonthaburi. Samples consisted of nasal, oral, or rectal swabs collected under the SARS-CoV-2 research project supported by Health Security Partners, the U. S. CDC, and the Thailand MoPH-U. S. CDC Collaboration (Table 2; Supplementary Table S2).

**Table 1:** Studied population of free-ranging *Macaca fascicularis*, in different parts of Thailand, March–November 2019.

Region	Province	No. of colonies	No. of samples
Central	Bangkok	1	26
	Lopburi	2	51
	Saraburi	1	25
	Nakhon Sawan	2	51
	Phetchabun	1	26
Northeast	Amnat Charoen	1	24
	Maha Sarakham	1	25
	Mukdahan	1	25
East	Chon Buri	4	100
West	Phetchaburi	3	75
	Ratchaburi	2	47
South	Krabi	2	50
	Narathiwat	1	18
	Phatthalung	1	25
	Satun	1	25
Total		24	593

**Table 2:** Studied population of exotic pets and small mammals presented by taxonomic order and location in three provinces of Thailand, May–September 2022.

Order	Animal hospital, Bangkok	Animal hospital, Nakhon Pathom	Garbage dumping site, Bangkok	Garbage dumping site, Nakhon Pathom	Garbage dumping site, Nonthaburi	Total
Lagomorpha	163	82	0	0	0	245
Primates	9	5	0	0	0	14
Carnivora	8	0	0	0	0	8
Rodentia	87	33	76	20	100	316
Erinaceomorpha	10	2	0	0	0	12
Diprotodontia	23	14	0	0	0	37
Eulipotyphla	0	0	22	0	0	22
Scandentia	0	0	0	1	0	1
Total	300	136	98	21	100	655

Swab samples were collected by trained veterinarians wearing full personal protective equipment, including gloves, masks, and coveralls. Gloves were changed frequently to prevent cross-contamination. Single-use sterile flocked nylon swabs were used, and each swab was immediately placed into 1 mL of phosphate-buffered saline (pH 7.4) or viral transport medium. Samples were maintained on ice during collection, transported to the laboratory at 4°C within 48 h, and stored at −80°C until use. Freeze–thaw cycles were strictly avoided to maintain sample integrity.

### Sample pooling

Swab samples were pooled by specimen type to maintain a consistent biological matrix and avoid potential cross-tissue interference. Each individual pool consisted of 4–5 swabs; 40–50 µL was collected from each swab and combined to achieve a final pool volume of 200 µL. This total volume of 200 µL was then used for DNA extraction, ensuring that each individual specimen was adequately represented in the diagnostic reaction.

### Viral DNA extraction and real-time PCR assay for MPXV detection

DNA was extracted from the pooled swabs using the genomic DNA mini kit (Geneaid Biotech Ltd., New Taipei City, Taiwan), according to the manufacturer’s instructions. In total, 553 pooled swabs were processed, with each DNA sample eluted in a final volume of 40 µL.

Target gene selection: To detect the MPXV genome in DNA samples, the *B7R* gene, which encodes a soluble interferon-gamma receptor, was selected as the diagnostic target because of its high conservation within the MPXV genome [45].

**Primer and probe design:** To ensure species specificity and prevent cross-reactivity with other orthopoxviruses, such as VARV or vaccinia virus, primers and probes were designed to target unique motifs within the *B7R* open reading frame [45]. The sequences were as follows:

MPXV_B7R_forward: 5′-ACGTGTTAAACAATGGGTGATG-3′

MPXV_B7R_reverse: 5′-AACATTTCCATGAATCGTAGTCC-3′

MPXV_B7R_probe: 5′-FAM-TGAATGAATGCGATACTGTATGTGTGGG-BHQ1-3′

**Reaction condition:** Assays were performed in a 20 µL total volume using the QuantiNova Probe RT-PCR Kit (Qiagen, Hilden, Germany). The reaction mixture comprised 5 µL of template DNA, 10 µL of 2× QuantiNova master mix, 0.4 µM of each primer and probe, 1 µL of QN ROX Reference Dye, and 2.8 µL of RNase-free water.

The thermal cycling profile for real-time PCR included initial denaturation at 95°C for 2 minutes, followed by amplification for 40 cycles at 95°C for 15 seconds and 58°C for 60 seconds.

Quality control and contamination prevention: Positive control, consisting of nucleotide design and synthesis from the partial genome of MPXV with 3.7 × 10^5^ copies/µL, and a negative control, consisting of nuclease-free water, were included in each run. To prevent contamination, pre- and post-PCR areas were physically separated, and aerosol-resistant pipette tips were used.

**Data analysis:** Fluorescence was captured using QuantStudio Software (Applied Biosystems, Waltham, MA, USA).

**Result interpretation:** A cycle threshold (Ct) value of ≤ 40 was defined as the cutoff for a positive result, consistent with standard diagnostic protocols [46, 47]. This cutoff is widely used in mpox surveillance to maximize sensitivity for low viral load samples. To mitigate the risk of false-positive results at late cycles, any sample with a Ct value of 34 or higher should be re-extracted and re-tested to ensure that no cross-contamination had occurred [48].

**Analytical sensitivity:** Original validation of *B7R*-targeted assays using the recombinant plasmid reported a limit of detection (LoD) of approximately 20 copies per reaction [45].

### Sample size and statistical power

In this study, a total of 1,248 archival animal samples collected across Thailand were tested to detect the presence of MPXV using real-time PCR. To ensure the reliability of potential negative findings, the sample size was evaluated based on its capacity to detect a pre-defined minimum threshold of pathogen prevalence.

In the event of zero positive detections, the 95% confidence interval (CI) for population prevalence was calculated using the rule-of-three (3/n), providing an upper bound for the maximum possible prevalence in the sampled population [49]. Furthermore, surveillance power was assessed as the probability of detecting at least one positive case under various low-level prevalence scenarios, such as 0.1%, 0.5%, and 1% [50]. Calculations were performed using the binomial distribution formula *Power* = 1 − (1 − *d*) *^n^*, where *d* represents the design prevalence and *n* represents the sample size [51]. For example, the probability that 1,248 samples would have yielded at least one positive result if the virus were actually present at a prevalence of 0.5% can be calculated as *Power* = 1 − (1 − 0.005) 1248.

### Statistical analyses

Microsoft Excel LTSC Professional Plus 2021 (Microsoft Corporation, Redmond, WA, USA; Version 2108) was used for data management. The prevalence of MPXV was calculated as the proportion of positive results among the total number tested and expressed as a percentage. Data were analyzed using descriptive statistics to estimate the current burden of MPXV and active viral shedding within the sampled population. Inferential statistics were not applied because the cross-sectional design of the study was intended to provide viral prevalence data rather than test specific comparative hypotheses.

## RESULTS

### Composition of the studied animal population

The studied population consisted of 29 species representing a total of 1,248 animals, including 593 cynomolgus macaques, 436 exotic pets, and 219 small mammals. The largest proportion of animals (47.5%, 593/1,248) comprised free-ranging *M. fascicularis*, whose colonies were located close to human habitations and communities in 15 provinces across Thailand (Table 1).

The second largest group consisted of exotic pets (34.9%, 436/1,248) presented to animal hospitals in Bangkok and an adjacent province (Table 2). Among exotic pets, rabbits *Oryctolagus cuniculus* belonging to the order Lagomorpha represented the majority (56.2%, 245/436), followed by rodents of the order Rodentia (27.5%, 120/436). The remaining animals (16.3%, 71/436) included species belonging to the orders Primates (bushbabies, marmosets, slow lorises, and a tamarin), Carnivora (wild cats, fennec foxes, ferrets, and raccoons), Erinaceomorpha (hedgehogs), and Diprotodontia (sugar gliders and a wallaby).

The final group consisted of small mammals (17.5%, 219/1,248) collected from garbage dumping sites, including rodents of the order Rodentia (89.5%, 196/219), shrews of the order Eulipotyphla (10.0%, 22/219), and a treeshrew of the order Scandentia (0.5%, 1/219) (Table 2). Among these, *Rattus tanezumi* and *Suncus murinus* were the most frequently identified species of rats and shrews, respectively. Detailed information on collected samples, including year, location, species, number, and sample types, is summarized in Supplementary Table S1 and Supplementary Table S2.

### Geographic distribution of sampled animals

**Figure 1:**
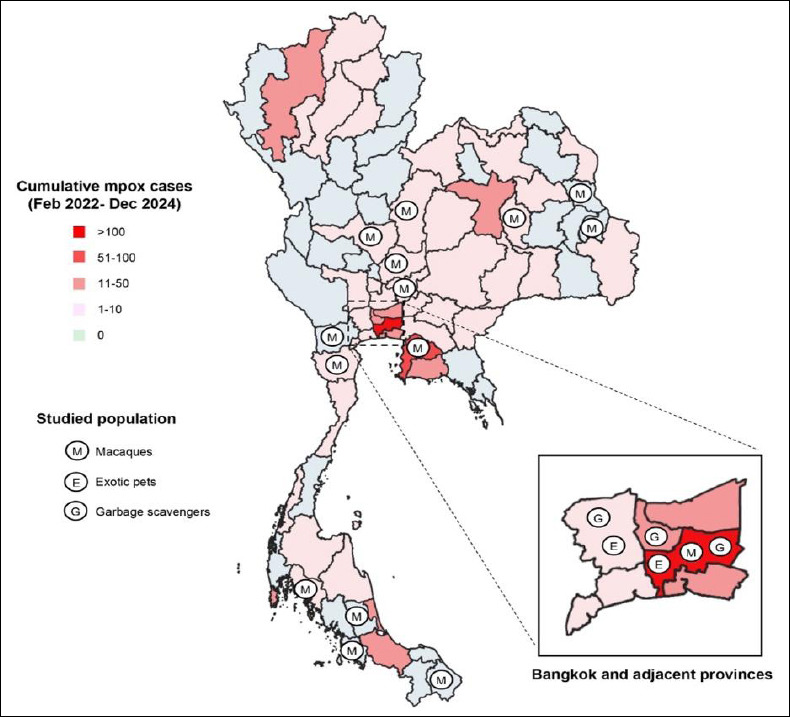
Map of Thailand showing the distribution of studied populations collected in 2019 (macaques) and 2022 (exotic pets and garbage-scavenging small mammals). The background map illustrates cumulative confirmed mpox cases reported between 2022 and 2024 using gradients of pink. Data source for mpox cases in Thailand: https://ddc.moph.go.th/monkeypox/ dashboard.php.

The distribution of studied animals covered several provinces where human mpox cases had been reported (Figure 1). The majority of animals originated from Central Thailand, particularly Bangkok, which was the main epicenter of the mpox outbreak in the country, with more than 450 cumulative confirmed cases reported [32].

Most exotic pets included in the study were obtained from an animal hospital in Bangkok (68.8%, 300/436), whereas the remaining animals (31.2%, 136/436) were derived from a hospital located in Nakhon Pathom province. Similarly, garbage-scavenging small mammals were collected from dumping site areas in Bangkok (44.7%, 98/219), Nakhon Pathom (9.6%, 21/219), and Nonthaburi (45.7%, 100/219). These two adjacent provinces also reported mpox cases, particularly Nonthaburi, which had the third highest cumulative number of confirmed cases in Thailand (50 cases as of 19 October 2025).

Among free-ranging macaques, nearly one-third of the animals (30.2%, 179/593) were sampled from colonies located in Bangkok and four additional provinces in the central region, including Lopburi, Saraburi, Nakhon Sawan, and Phetchabun, all of which reported small numbers of mpox cases. Additional macaque populations were sampled from the Northeastern region (12.4%, 74/593), eastern region (16.9%, 100/593), western region (20.6%, 122/593), and southern region (19.9%, 118/593).

Some macaque colonies were located in provinces affected by the outbreak, including Maha Sarakham (northeast), Chon Buri (east), Phetchaburi (west), and Krabi (south). Among these, Chon Buri reported the second highest cumulative number of confirmed cases in Thailand (85 cases as of 19 October 2025), whereas the remaining provinces reported at least one confirmed case since the outbreak began.

### MPXV detection by real-time PCR

DNA extracted from swab samples of all 1,248 animals was tested for MPXV using real-time PCR targeting the *B7R* gene. All samples were negative for MPXV DNA, with the cutoff for a positive result defined as Ct ≤ 40 cycles. No inconclusive or borderline Ct values were observed. All samples produced a “not detected” result, and no late-cycle amplification signal below the threshold was recorded.

These findings indicate that MPXV DNA was not detected in any of the sampled animals at the time of collection.

### Prevalence estimation and statistical power analysis

All 1,248 animals tested negative for MPXV DNA by PCR. Based on this sample size, the upper limit of the 95% CI calculated using the rule-of-three indicated that the prevalence of MPXV in the studied population was <0.24%.

Power analysis demonstrated that a sample size of 1,248 provided a 99.81% probability of detecting at least one positive case if the true prevalence of MPXV in the population was 0.5%. The study therefore achieved the standard 95% confidence threshold for excluding a prevalence of 0.24% or higher.

In addition, statistical power remained above 70% for detecting a prevalence as low as 0.1%, indicating that the absence of positive results represents a statistically robust lack of widespread viral circulation in the studied animal populations.

## DISCUSSION

### Baseline evidence of MPXV absence in studied animals

The current study examined the presence of MPXV DNA in archival swab samples from wild animals and exotic pets collected in 2019 and 2022. A total of 1,248 swab samples derived from 593 cynomolgus macaques, 436 exotic pets, and 219 small mammals were found negative for MPXV DNA. Although no viral DNA was identified from the animals during the study period, our finding provides baseline evidence of MPXV absence in wildlife and exotic animal populations in Thailand prior to and during the early phase of the global 2022 outbreak. Evidence of absence demonstrated through well-designed studies is critical for risk assessment, surveillance prioritization, and policy decision-making, particularly in the context of emerging zoonotic threats.

### Novelty and significance of the study

The novelty of this study lies in the use of pre- and early-outbreak archival samples, multi-population risk-based animal selection, and focus on spillback prevention in a non-endemic country. To our knowledge, this is the first nationwide molecular surveillance study in Southeast Asia assessing MPXV absence in wildlife, exotic pets, and garbage-scavenging mammals during the 2022 global outbreak. A model of One Health surveillance framework that integrates wildlife, exotic animals, and environmental exposure pathways demonstrated by this study can be adapted by other non-endemic countries during emerging zoonotic outbreaks.

### Mpox situation in Thailand and implications for surveillance

Historically, Thailand has no record of human and animal cases of mpox. However, the recent largest outbreak in 2022 affected more than 120 countries worldwide, of which at least 115 countries are located outside endemic regions where mpox had never been previously reported, including Thailand [16, 52]. The ongoing global outbreak caused more than 163,000 cases with more than 420 reported deaths as of 30 September 2025 [16]. Thailand reported the first human case in July 2022 in a Nigerian man who had entered Thailand since October 2021 [53]. From the first case identified, the Thai population has been infected with MPXV, resulting in more than 930 confirmed cases and 13 deaths so far [16, 32]. Similar to other non-endemic countries, the MPXV outbreak in Thailand has been classified as a subclade IIb strain. However, the first case of subclade Ib was confirmed in Thailand in August 2024, in which the case had traveled from an African country [54]. No additional case infected with subclade Ib had been reported in Thailand until now.

Based on genome sequence data, the 2022 outbreak has clearly occurred through the exportation of MPXV from affected areas of Africa to other countries [55, 56]. The virus has been spread mainly through sexual or intimate contact [10, 11, 52]. However, the cryptic transmission in human and animal hosts that probably occurred prior to 2022 in a non-endemic country should be taken into consideration [55, 57]. The inclusion of archival animal samples collected prior to the 2022 global outbreak represents a rare opportunity to assess cryptic circulation of MPXV before widespread human transmission in a non-endemic region. Additionally, a wide variety of animal species can become infected with MPXV via direct contact or fomites [58]. Infected animals, especially wildlife, can become new reservoirs of MPXV in unaffected geographic regions [34, 58]. Although the specific reservoir species of the virus have not been identified, several potential animal reservoirs, such as rodents and non-human primates, may harbor the virus, leading to occasional spillover events to humans [55, 57]. Thus, monitoring and surveillance of animals at high-risk of viral infection, particularly those in close contact with infected persons, are necessary to detect and possibly prevent the establishment of animal reservoirs [34].

### Rationale for inclusion of the selected animal populations

A convergence of extensive urban wildlife populations, widespread exotic pet ownership, and close human–animal contact would make a non-endemic country like Thailand a high-risk interface for the establishment of MPXV and an ideal sentinel setting for early spillback surveillance. We attempted to include potential animal populations for MPXV detection in the pre- and early-2022 mpox outbreak. This study uniquely integrates three distinct animal populations representing direct contact, trade-associated, and environmentally exposed pathways, enabling a comprehensive assessment of MPXV spillback risk across the human–animal–environment continuum.

Free-ranging *M. fascicularis* were included in the study because non-human primates are susceptible to MPXV infection. Moreover, Thailand has a serious problem of macaque overpopulation in several cities, especially tourist destinations [59]. All macaque populations in the study lived close to human settlements, placing them at high-risk of virus transmission through direct human contact.

Another target population for MPXV detection was exotic pets, in which all exotic animals in this study were owned pets and several species can be infected with MPXV. Exotic pets are considered wild species rather than domesticated species [60]. Moreover, exotic animals are often non-native or non-indigenous, and some are imported from other countries [61]. Without proper pathogen screening, imported animals can facilitate the transmission of MPXV, and the global trade of these animals creates environments where close, cross-species contact significantly amplifies viral transmission and spreads infectious diseases across borders. According to evidence from the 2003 outbreak in the U. S., MPXV was transmitted through imported rodents from Africa, in which prairie dogs and several native species became infected following close contact with those imported animals [29]. In this study, the exotic pets, although lacking a history of exposure to human mpox cases, were nonetheless considered a potential source of virus infection and transmission.

The last population studied for the presence of MPXV comprised small mammals scavenging around garbage dumping sites. In urban areas of Thailand, landfills are the most common form of waste disposal. Household waste is collected and transported to landfill or garbage dumping sites for disposal. Most landfill sites are open areas that cannot limit animal access, resulting in potential risk of virus transmission to animal scavengers [62]. Generally, poxviruses can persist from weeks to several months, depending on surface materials and environmental conditions [33, 63–65]. A recent study demonstrated that vaccinia virus was stable on different surfaces such as plastic, towel, paper, and glass, and the virus could persist much longer at low temperature at 4°C and −20°C [66]. Exposure to contaminated environments can potentially transmit infectious viruses. Hence, garbage-scavenging animals could be at high-risk of contracting disease through environmental exposure, particularly to domestic waste originating from settings where mpox cases have been reported. In this study, small mammals representing garbage-scavenging species were sampled from garbage dumping sites in Bangkok and two additional provinces with confirmed mpox cases. However, no link was identified between the animal collection sites and the human case locations or contaminated waste.

### Susceptibility of non-human primates and rodents

In the group of non-human primates, *M. fascicularis*, bushbabies (*Galago* sp.), marmosets (*Callithrix* sp.), slow lorises (*Nycticebus* sp.), and tamarin (*Saguinus midas*) were included in our study, in which *M. fascicularis* were free-ranging animals while the others were represented in the group of exotic pets. Based on animal experiments, *M. fascicularis* has been found to be highly susceptible to MPXV infection through intravenous exposure [67, 68]. The infected macaques showed clinical signs resembling human mpox, including vesiculopapular rash, fibrinonecrotic pneumonia, lymphadenopathy, and death within 9–17 days post-infection [67, 68]. *M. fascicularis* exposed intranasally or via aerosol to MPXV have also exhibited symptoms associated with mpox [69, 70]. Marmosets *Callithrix* sp. were another non-human primate that has been used as an animal model for mpox study, but their clinical signs resembled smallpox rather than mpox [71].

Potential reservoirs among rodent species were also investigated for MPXV in the current study. Thirteen different rodent species were included, in which 10 species of rodents (squirrels, prairie dogs, dumbo rats, bamboo rats, woodland dormouse, guinea pigs, chinchillas, and hamsters) were represented in the group of exotic pets and four species of rats (Pacific rats, Norway rats, Oriental house rats, and unidentified rats) were represented in the group of garbage-scavenging animals. All rodent species can possibly be infected with MPXV, especially prairie dog *Cynomys* sp., which was known to be a potential animal reservoir for MPXV [72]. The 2003 outbreak investigation in the U. S. demonstrated that black-tailed prairie dogs *Cynomys ludovicianus* that had contact with infected African rodent species were accidentally infected, became sick, and died during the outbreak [31]. Experimentally infected prairie dogs have exhibited clinical characteristics resembling human mpox disease. Earlier studies in the prairie dog model have confirmed high levels of MPXV shedding via oral, nasal, ocular, and rectal routes, as well as animal-to-animal oral, respiratory, and mucosal transmission [73–75]. Therefore, this species has been widely used as a model to study the pathogenesis and transmission of MPXV [76, 77]. Another susceptible species for MPXV infection is an African dormouse species *Graphiurus* sp., in which *Graphiurus murinus* was present in our exotic pet population. Previous studies of mpox have used Kellen’s dormouse *Graphiurus kelleni* as an animal model. Kellen’s dormouse has been shown to be highly susceptible to intranasal infection with clade I MPXV, and infected animals could shed the virus in nasal washes, suggesting that this species can shed infective virus into the environment via nasal secretions [78].

### Other susceptible mammalian species

Other mammal species in our studied population, such as hedgehogs, rabbits, and shrews, are known to be susceptible to MPXV infection. Hedgehogs and rabbits were represented in the exotic pet population, while shrews were represented in the garbage-scavenging animal population in our study. MPXV infection under natural conditions has been evident in hedgehogs and shrews. Following the 2003 MPXV outbreak in the U. S., hedgehogs *Atelerix* sp. sharing housing areas close to infected African rodents tested positive for MPXV. Further investigation confirmed the presence of viral DNA in various tissue samples of the infected hedgehogs [29]. On the other hand, antibodies against OPXV have been detected in shrews from Zambia and the DRC at rates of 33.3% (14/42) and 1.2% (1/84), respectively [79, 80]. For rabbits, this species has been tested for MPXV infection in the laboratory. Susceptibility depended on the inoculation method and the animals’ age [81]. Infected rabbits exhibited acute illness with a generalized rash when challenged via the intravenous route, but not via oral inoculation [81]. Moreover, young rabbits have been shown to be more susceptible to MPXV infection than adult rabbits [81]. However, no evidence of natural MPXV infection in rabbits has been reported.

### Effect of sample pooling and interpretation of negative findings

Sample pooling is a widely recognized strategy for large-scale surveillance, particularly when expected pathogen prevalence is low (typically <5%–10%). In this study, pooling 4–5 swabs per reaction was utilized to maximize laboratory throughput and optimize reagent consumption. While pooling increases population-level sensitivity by allowing the screening of a larger number of animals, it introduces a potential reduction in individual diagnostic sensitivity due to the dilution effect. Previous studies have shown that pooling five samples typically increases the Ct value by approximately 2.0 to 2.6 cycles [82]. Consequently, the risk of false-negative results is highest for specimens with low viral loads (e.g., Ct > 34), as the diluted target may fall below the assay’s LoD [83, 84]. However, for small pool sizes (≤ 5), sensitivity for moderate-to-high viral loads remains high (90%–100%), whereas sensitivity for low viral loads may decrease significantly [85].

Although real-time PCR results from this study indicated an absence of MPXV viral DNA in all tested animals, these negative results should be interpreted with caution. Potential false negatives could have resulted from suboptimal sample collection timing, the sample pooling effect, or poor sample quality. While PCR provides definitive evidence of active infection, it offers only a temporal snapshot of viral presence within the sampled animals. Our reliance on molecular detection means that prior infectious shedding or past exposure within these populations cannot be ruled out. Furthermore, as this study focused exclusively on PCR detection of MPXV DNA, we did not assess antibody status, viral infectivity, or transmission potential among sampled animals. To address this critical gap in understanding long-term viral circulation, particularly for archival samples collected in 2019, future research should prioritize serosurveillance. Implementing antibody-based screening alongside molecular detection is essential to map the full extent of past exposures and identify potential silent reservoirs in non-endemic regions.

### Practical recommendations for One Health surveillance

Based on our findings and the documented risks, we propose actionable recommendations for a robust One Health surveillance framework in non-endemic regions by focusing on three aspects:

Integration with human surveillance: Establish formal data-sharing protocols between public health and veterinary health agencies to facilitate rapid temporal and spatial correlation of emerging cases in both populations [86].

Pet trade regulation: Implement mandatory health screening and certification programs for all imported exotic mammals, particularly rodents and non-human primates known to be susceptible to orthopoxviruses, to prevent disease introduction via trade routes [87].

Wildlife–urban interface monitoring: Develop standardized, long-term monitoring programs for key sentinel species (e.g., macaques and urban-dwelling small mammals) in high-contact areas, using both molecular and serological methods to track viral circulation baselines and detect emergence events [88].

### Study limitations

This study has several limitations regarding the sampled animal populations and the diagnostic methodology employed. First, the low number per animal species limited virus detection and may not reflect the true prevalence of the disease in individual animal species. Furthermore, the study lacked molecular species confirmation (e.g., DNA barcoding of *cytB* or *COI* genes) for a subset of the sampled rodents, particularly those classified as unidentified rats. While morphological keys are standard for field studies, they may fail to distinguish between cryptic species common in Southeast Asia [89, 90]. This limits our ability to make definitive ecological inferences regarding species-specific reservoir competence, as different species within the same genus may vary in their capacity to maintain or transmit MPXV.

Second, the samples used in this study were opportunistically repurposed, which may affect the validity of the results. In addition, the period of sample collection and the habitation areas of the studied animals may not have been entirely relevant to the presence of human cases in the country. Nevertheless, samples collected prior to the outbreak could provide baseline information on the virus in potential animal reservoirs, particularly in a non-endemic region. Although this study demonstrated geographic overlap between animal sampling sites and human mpox cases, we could not establish a precise temporal linkage between animal sampling and human epidemiological trends. The pre-outbreak samples were collected in 2019, prior to the first laboratory-confirmed human mpox case in Thailand; therefore, no temporal overlap existed for this cohort to evaluate potential spillover or spillback events. Regarding the early-outbreak samples collected in 2022, while this period coincided with the emergence of the global outbreak, high-resolution temporal and spatial data for human cases in the specific sampling provinces were not publicly available to allow direct correlation. Consequently, we could not determine whether animal sampling exactly preceded or followed local peaks in human cases, which limits our ability to definitively assess the immediate risk of anthropozoonotic spillback during that period.

Third, single-time-point sampling of an individual hindered the ability to detect the virus during active infection in animal reservoirs because of the rapid clearance of the virus from mucosal surfaces and major organ systems [80, 91]. This is consistent with the previous report that viral shedding could be observed only briefly, between 5 and 22 days, in the African rope squirrel model [92]. Hence, repeated sampling is required to rule out infection in animal reservoirs.

Fourth, sample pooling may lead to a loss of diagnostic sensitivity due to dilution of low-titer viral DNA. Pooling dilution may result in underestimation of viral presence when individual viral loads are near the detection limit [83, 84].

Fifth, in the absence of serological testing, this study could not identify prior exposures or infections in the population. Because our methodology relied exclusively on PCR, which detects active viral shedding, we cannot rule out the possibility that MPXV circulated in these populations prior to sampling. This is particularly relevant for the archival pre-outbreak samples collected in 2019; animals may have cleared the virus before sampling, leaving no detectable DNA despite a history of infection.

Lastly, the lack of environmental sampling, such as testing wastewater, bedding, or soil/fomites at scavenging sites, may have obscured broader indicators of viral circulation within the study area. Environmental surveillance is a critical tool for detecting pathogens in a community before individual clinical cases emerge [93–96]. Future research should prioritize these methods within a One Health framework to better understand viral persistence and transmission dynamics in these settings.

## CONCLUSION

This study investigated the presence of MPXV DNA in 1,248 archival swab samples collected from free-ranging cynomolgus macaques, exotic pets, and garbage-scavenging small mammals in Thailand during the pre-outbreak (2019) and early-outbreak (2022) periods. All tested samples were negative for MPXV DNA by real-time PCR targeting the *B7R* gene. Based on the sample size, the upper limit of the 95% CI indicated that the prevalence of MPXV in the studied animal population was <0.24%, and statistical power analysis demonstrated a high probability of detecting the virus if it had been circulating at low prevalence. These findings provide the first nationwide baseline evidence supporting the absence of detectable MPXV in selected wildlife and exotic animal populations in Thailand before and during the early phase of the 2022 global outbreak.

From a practical perspective, the results suggest that, despite widespread human cases during the global outbreak, there was no indication of established animal reservoirs in the investigated populations at the time of sampling. This information is important for public health authorities because the establishment of permanent reservoirs in wildlife or exotic animals could allow the virus to persist independently of human transmission, leading to recurrent outbreaks. The risk-based selection of animals living in close proximity to humans, including urban macaques, exotic pets, and garbage-scavenging mammals, strengthens the relevance of the findings for spillback prevention strategies in non-endemic regions.

A major strength of this study is the use of archival samples collected before and during the outbreak, which enabled evaluation of possible cryptic circulation of MPXV prior to widespread human transmission. In addition, the inclusion of multiple animal populations representing different exposure pathways, together with nationwide geographic coverage, provides a comprehensive One Health-based surveillance model that integrates wildlife, exotic animals, and environmental interfaces. The relatively large sample size and statistical evaluation of surveillance power further support the reliability of the negative findings.

Nevertheless, the absence of viral DNA should not be interpreted as definitive proof that MPXV has never circulated in animals in Thailand. Limitations such as single-time sampling, pooling of specimens, absence of serological testing, and lack of environmental sampling may have reduced the ability to detect low-level or past infections. Therefore, future studies should incorporate longitudinal sampling, serosurveillance, environmental monitoring, and molecular species confirmation to better understand the long-term ecology of MPXV in non-endemic settings. Expanding surveillance to additional wildlife species and integrating animal data with human epidemiological information will be essential to detect early spillback events and prevent the establishment of new reservoirs.

In conclusion, the present study provides baseline molecular surveillance evidence that MPXV was not detected in selected wildlife, exotic pets, and urban scavenging mammals in Thailand prior to and during the early phase of the 2022 outbreak. Continued One Health-based surveillance, particularly in regions with intense human–animal interaction and active wildlife trade, is essential to prevent reservoir formation and to support early detection and control of emerging zoonotic diseases.

## DATA AVAILABILITY

The supplementary data can be made available from the corresponding author upon request.

## AUTHORS’ CONTRIBUTIONS

TC, MT, WP, and NC: Conceptualization, writing–original draft preparation, and writing–review and editing. TC, NK, SC, LS, SS, and WP: Methodology. MT, WP, and NC: Validation. NK, MT, SC, LS, SS, and WP: Formal analysis. TC, NK, MT, SC, LS, SS, and WP: Investigation. TC, NK, MT, SC, and WP: Data curation. NC: Supervision and project administration. All authors have read, reviewed, and approved the final manuscript.
